# Suggestions for the Treatment of Insomnia

**Published:** 1911-09-16

**Authors:** C. Willett Cunnington


### The General Practitioner's Column.

### SUGGESTIONS FOR THE TREATMENT OF INSOMNIA.

By C. WILLETT CUNNINGTON, M.B., B.C. Cantab.

Insomnia is a condition which is met with in varying degrees almost daily in general practice, yet it is one which is treated only too frequently in some routine fashion, and often enough baffles treatment altogether.

### Varieties of Insomnia.

The problem of "getting a patient to sleep" is sometimes obstinately difficult, sometimes dangerously easy, and this is apt to be the case when the only weapon employed is some sort of hypnotic. The purpose of this paper is to suggest the methods of approaching and dealing with this problem. There is no routine solution, because each case needs careful investigation as to the cause of the condition before the appropriate treatment can be arrived at. For this purpose it is useful to make a rough division of cases into two main groups: (1) Those in which the insomnia is a condition dependent on some definite disorder, whether of an organic or of a functional nature; (2) those in which there appears to be no such primary disorder. We may refer to the one group as secondary and to the other as primary insomnia. There are many diseases of which sleeplessness is a marked feature, but for the purpose of this paper we may set aside such cases, seeing that the symptom in question forms only a small part in the problem of treatment. We may, then, disregard the insomnia occurring in acute fevers or produced by severe physical pain, and consider in this category only those patients whose most prominent symptom is sleeplessness. It is necessary, first of all, to decide whether a given case belongs to the primary or secondary group. This entails an investigation, especially of the nervous, vascular, and urinary systems, putting them in their order of importance.

### Nervous Causes.

Of the nervous system diseases, such as commencing general paralysis, chorea, cerebral tumour, and mental diseases, may be mentioned as typical examples. In some of these and similar nervous diseases the insomnia is due to pain; in others to excessive nerve irritability. Reference must be made to that functional nervous disease or symptom-complex described as neurasthenia. Here inability to sleep may be the chief complaint of the patient, the other symptoms being elucidated only by questions. It is a very familiar condition, and only needs mention, because in some patients the insomnia is so marked that it demands primary attention. These need absolute confinement to bed, forced feeding, and, for a time at least, a sleeping draught at night. Paraldehyde in drachm doses is particularly serviceable.

### Circulatory Causes.

Diseases of the vascular system as causes of insomnia are more important because more often overlooked. As soon as cardiac compensation begins to fail, sleeplessness becomes noticeable; but it may be present even when compensation appears good. This is particularly the case in aortic regurgitation. Such a patient will describe his sleep as a matter of "fits and starts," and his short snatches of slumber are liable to be troubled with harassing dreams.

A more common vascular cause of insomnia is arterio-sclerosis, and this is often overlooked. A typical example would be the over-nourished, strenuous man of business who finds, when he has reached the "critical period" of the fifties, that "his nights are not as good as they used to be." He ascribes those hours of wakefulness, occurring from 2 A.M. onwards, to business worries, pressure of work, and the like, and the suggestion put forward by him is often too readily accepted by his medical attendant, who is led to recommend a bracing holiday as a cure. The patient, snatching a fortnight from the office, hastens to Swiss mountains to be "braced up," and adds further strain to his brittle arteries.

Similarly the patient with chronic albuminuria may come complaining only of sleeplessness. The danger of treating these vascular and renal cases in a routine fashion by soporifics is obvious enough. They are suffering probably from defective cerebral circulation or defective excretion of nitrogenous metabolised products, and need treatment on those lines.

Often enough the man with thickened vessels complains that of an evening after dinner he will doze in his chair, only to find himself unable to sleep later on when he retires to bed. The warmth of the fireside relaxed his peripheral arterioles, which contracted again in the cold of the bedroom.

### Primary Insomnia.

Suppose a patient presents no evidence of disease of the systems mentioned, and we are compelled, a little reluctantly, to classify him as a case of primary insomnia. In reality this signifies that the exciting cause is of a psychological nature; there are many such, some obvious, some less so.

Expressed aphoristically, in business men troubles of work, in married women troubles of the home, and in young people troubles of the heart, these rob the sufferers of sleep. People will tell their doctor of the existence of business worries, but on more intimate matters they are reticent, and questions have to be put. One finds that all these private troubles are of two types: anxiety as to the future or unhappiness as to the past. It is the duty of a doctor to question, without prying, for he may be able to give advice in the former and comfort in the latter. To these patients words of wisdom will be more valuable than even the most ingenious prescription. A broken heart, for instance, is not to be cured by coal-tar compounds. There remain, however, a number of patients who have nothing on their minds and yet cannot sleep. In some there is a family tendency, in others a habit has grown, perhaps, from small beginnings. These are exceedingly difficult to manage successfully. Before resorting to drugs there are a number of small points worth attention. The doctor must be satisfied that every material factor is favourable for obtaining sleep. It is odd that people will neglect to see that their bedrooms are absolutely dark and quiet at night. Cold feet will keep people awake, and they have often learnt the value of hot-water bottles.

In habit cases it may help to advise a change of bedroom or even of house for a time. The evening meal is an important matter, but no general rule can be laid down, as one person will be awake because his stomach is empty and another because it is otherwise. The late dinner of the full-blooded, over-nourished man needs curtailing, while the thin, dyspeptic, nervous woman requires more nourishment. For them some easily digested milk food the last thing at night is useful. Tea and coffee are best avoided, especially in the latter part of the day. On theoretical grounds alcohol is best abstained from, but in people over fifty it is seldom wise to change a habit which may have become second nature. The popular fancy for a "night-cap" has its obvious objections.

Attention must be paid to the proper ventilation of the patient's bedroom, and he should be urged to spend as many hours as possible out of doors each day. Extra pillows will help the heart cases, and this device may be of service to some others as well.

A few words may be added about those unfortunates who perhaps themselves recognise that their insomnia is due to "the slings and arrows of outrageous Fortune." They are too often familiar with the cheerless comment that "the case is not a medical one." On the contrary, it is to such people especially that useful advice and help can be given, and it is important that they should be convinced that help can be given by their doctor. Generally they will describe their nights as "fairly good until 2 A.M." After that hour they lie awake and brood over their troubles. Most of us have had personal experience of that worst of all nightmares "the 2 A.M. worry." They should be urged not to lie thinking, but to read on waking. Let them have books at their bedside to which they can turn. Even the selection of the volumes is not outside the province of a wise medical attendant. To replace the sleep missed at night these patients should lie down for an hour or so after lunch. The afternoon nap is a habit to be encouraged. Sometimes the régime of the day needs entire change; one has to remember that sorrow is softened by distractions, and here a change of scene is helpful, while anxiety is not so readily relieved by such methods.

Lastly, and it should always come lastly to the doctor's mind, is the matter of drugs. A soporific may be regarded as an evil, yet in some cases as a necessary one. There is nothing safer, especially in primary insomnia, than a dose of bromide at bedtime, reinforced, if necessary, by the addition of chloral. The doctor should aim at reducing the dose insensibly as sleep is regained. Veronal is at present very fashionable, much too fashionable. Seldom is it necessary to give a larger dose than seven grains. It acts quickly and leaves no ill after-effects, except the temptation to make of it a "friend on the dressing table." Sulphonal, which acts more slowly, should be given in the afternoon. Most hypnotics tend to constipate, and therefore a saline aperient in the morning is desirable.

The patient with high blood-pressure will find drugs to relax the arterioles helpful. These are the people, if any, who benefit from alcohol at bedtime. Whatever drug is used its name and amount should, if possible, be concealed from the patient's knowledge, and the dose steadily diminished as soon as possible.

